# Canada’s National Advisory Committee on Immunization (NACI) in 2025: Celebrating 60 years of service, a decade of change, and a dynamic future ahead

**DOI:** 10.14745/ccdr.v52i03a01

**Published:** 2026-03-31

**Authors:** Matthew Tunis, Robyn Harrison, Kaeli Ramotar, Ashleigh Tuite, Christina Jensen, Krista Wilkinson, Kelsey Young, Joseline Zafack, Marina Salvadori, Adrienne Stevens, Vinita Dubey, Erin Henry

**Affiliations:** 1 Centre for Immunization Surveillance and Programs, Public Health Agency of Canada, Ottawa, ON; 2 Division of Infectious Diseases, Department of Medicine, University of Alberta, Edmonton, AB; 3 Alberta Health Services, Edmonton, AB; 4 Department of Pediatrics, McGill University, Montréal, QC; 5 Toronto Public Health, Toronto, ON; 6 Toronto Dalla Lana School of Public Health, University of Toronto, Toronto, ON

**Keywords:** immunization, vaccines, NACI, advisory committee, equity, Canada, public health policy

## Abstract

Canada’s National Advisory Committee on Immunization (NACI) marked its 60^th^ anniversary in 2024, representing six decades of reliable advice supporting Canada’s immunization programs. Over the past decade, NACI expanded its mandate to include ethics, equity, feasibility, acceptability, and economic considerations, while adapting its methods to align with international standards and responding to urgent public health needs such as the COVID-19 pandemic. Enhanced collaboration with provinces, territories, Indigenous partners, and global peers has strengthened both the relevance and reach of NACI guidance. With an expanding vaccine landscape, NACI continues to evolve as a trusted national and global resource supporting equitable, evidence-informed immunization policy and practice in Canada.

## Introduction

Canada’s National Advisory Committee on Immunization (NACI) has been providing independent advice on immunization to the Government of Canada since 1964 ((1)). The committee is aligned with the definition of a National Immunization Technical Advisory Group (NITAG) established by the World Health Organization (WHO). However, as one of the longest standing vaccine advisory committees in the world, NACI significantly predates the global alignment of NITAGs ((2)). The year 2024 marked the 60^th^ anniversary of NACI. This article, written in celebration of NACI’s 60 years of remarkable service in Canada, reviews the past decade, a period marked by many significant developments to the committee’s mandate, methods, and outputs during both a pandemic response and routine vaccine programs. It also looks ahead to an increasingly expansive vaccine landscape, new collective challenges and opportunities to enhance individual health and strengthen efficiencies in Canada’s national, provincial and territorial health systems.

### Mandate

**Figure 1:**
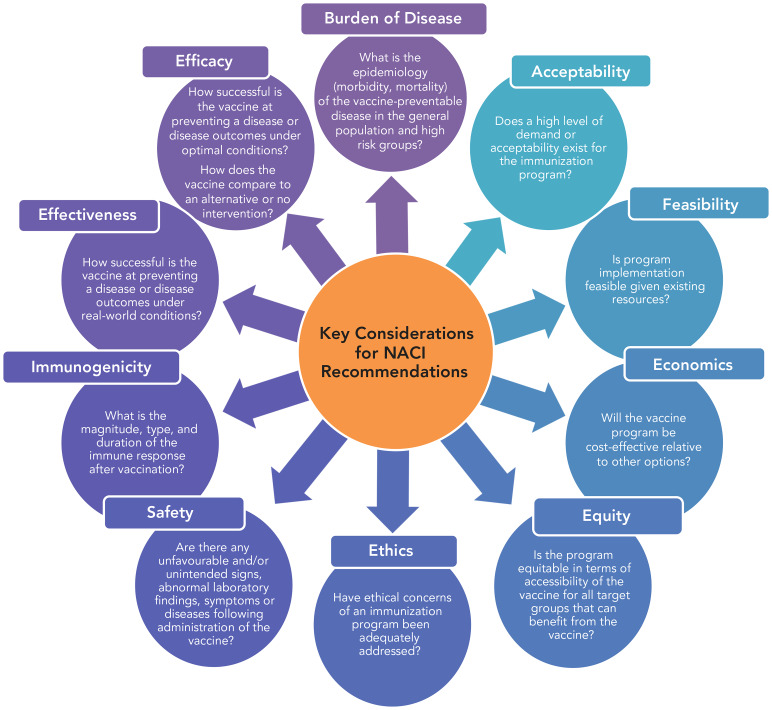
National Advisory Committee on Immunization Decision Framework Abbreviation: NACI, National Advisory Committee on Immunization

The past decade has seen a significant expansion of the NACI mandate, along with several adaptations to reflect the increased complexity of the Canadian immunization program environment. Canada has had a National Immunization Strategy (NIS) in place since 2003 ((3)). In 2016, the NIS was renewed by the federal government and provincial/territorial Deputy Ministers of Health to include several new objectives, notably, to ensure that “Canadians have timely and equitable access to immunization,” to be accomplished by expanding the mandate of NACI to enhance the timeliness and scope of its recommendations ((4)). This NIS objective led Dr. Theresa Tam, the Assistant Deputy Minister accountable for NACI at the Public Health Agency of Canada (PHAC) at the time, to announce in 2016 that an expanded mandate for the committee would be developed and fully implemented by 2019. The expanded mandate included five new programmatic factors drawn from the Erickson, De Wals, Farand analytical framework for immunization programs in Canada ((5)): economics, ethics, equity, feasibility, and acceptability (**Figure 1**).

Prior to 2016, the federal/provincial/territorial Canadian Immunization Committee (CIC) would often publish program assessment statements as part of a two-step process to complement the NACI scientific assessment, but this took several years and could result in delayed decision-making for implementation. Under NACI’s expanded mandate, programmatic factors are captured during the assessment of disease burden and vaccine characteristics in order to provide provinces and territories with a more complete package that facilitates timely program implementation decisions. In 2025, under the current model, the CIC (representing the provinces and territories) is now engaged at three key touchpoints: first when the NACI topic scope is being defined; second, during feasibility assessments in the course of guidance development; and third, in reviewing draft recommendations before they are finalised by NACI.

The NACI Secretariat at PHAC grew since 2016 to support the expanded NACI mandate, including experts in vaccine modelling, health economics, and social sciences. NACI has been very active in providing emergency guidance on vaccines for outbreak responses (including the COVID-19 pandemic, mpox, and measles). Several adaptations were necessary throughout the pandemic to achieve an emergency response model that adapted to the urgency and evolving scientific data during these situations ((6)).

### Ethics, equity, feasibility, acceptability

To implement these new programmatic elements throughout 2016–2019, the NACI Secretariat at PHAC and NACI consulted with CIC to develop evidence-based, peer-reviewed frameworks for assessment and inclusion of ethics, equity, feasibility, and acceptability (EEFA) elements ((7)). Each programmatic element is addressed by a matrix within the overall EEFA framework. An illustrative example is NACI’s use of the equity matrix to identify key populations for prioritization of COVID-19 vaccines ((8)). In practice, the EEFA framework requires ongoing engagement of provincial and territorial vaccine program experts through CIC. Equity considerations have led to specific recommendations. Tangible examples from NACI’s EEFA framework include the prioritization and strong COVID-19 vaccine recommendations for racialized and marginalized populations (2021–present). In 2020, equity considerations also guided early vaccine prioritization for people living with disabilities and for those whose living or working conditions placed them at higher risk of infection with disproportionate consequences (e.g., correctional facilities, agricultural or meat production/packing facilities, congregate living settings, and individuals who are unhoused). Other examples are the inclusion of COVID-19 vaccine for people in or from First Nations, Inuit, and Métis communities (2020–present), strong recommendations for use of respiratory syncytial virus (RSV) prophylaxis for all infants in communities where medical transport can be long and complex (2024–present), and recommendations for pneumococcal conjugate vaccines in adults younger than 65 years of age with risk factors relating to medical conditions or environmental/behavioural factors such as being unhoused (2023–present).

When ethical considerations are particularly complex, NACI has the opportunity to consult the Public Health Ethics Consultative Group (PHECG), an external advisory body to PHAC, for a detailed scenario-based ethical analysis. Throughout the COVID-19 pandemic, NACI consulted PHECG on 13 occasions from 2020 to 2022 on COVID-19 topics, such as extended intervals, population prioritization, boosters, and off-label use in paediatrics. Since then, NACI has continued to benefit from PHECG advice on topics, such as prioritizing doses of mpox vaccine, human papillomavirus (HPV) schedule reductions, and RSV vaccination during pregnancy.

Importantly, NACI continues to issue off-label or expanded use recommendations to improve equity when supported by a public health ethics analysis. This is sometimes accomplished by routine EEFA assessments; in other cases, NACI will request detailed advice from PHECG when the benefits and risks or knowns and unknowns are closely balanced.

### Economic evidence

In addition to EEFA programmatic factors, economic evidence was also added into the scope of NACI decision criteria, consistent with practices used by other long-established advisory committees in the United States of America (USA), United Kingdom (UK), Germany, and elsewhere. To support this change, from 2019 to 2023, PHAC and NACI worked with experts to develop two complementary tools: guidelines for the economic evaluation of vaccine programs in Canada ((9)), aimed at producers of economic evidence, and an interpretation guide ((10)) to help readers of NACI statements understand the economic evidence summaries.

With the development of these guidelines and processes, NACI began routinely incorporating economic considerations into their guidance. NACI first determines if economic evidence is needed, typically through a prioritization exercise that incorporates input from NACI members and provinces and territories through the CIC ((11)). Canada does not use a cost-effectiveness threshold for vaccine decisions, and not every NACI statement will include economic evidence ((11)). For example, several emergency rapid response statements issued by NACI on COVID-19 and mpox did not require economic analyses to support deployment decisions from federal stockpiles. If economic evidence is needed, the approach is tailored based on the specific information required for the recommendation. This may involve conducting a systematic review of economic evaluations (sometimes provided by Canada’s Drug Agency, according to PHAC’s request and specifications), developing a new model-based economic evaluation, or conducting a multi-model comparison that may include both independent and industry-sponsored models. **Table 1** identifies which NACI statements over the last decade have included health economic evidence, including seven in the most recent year (2024).

**Table 1:** Inclusion of economic evidence in National Advisory Committee on Immunization statements, 2014–2024^a^

Year	Statement topic	Statement title	Economic evidence used
2018	Herpes zoster (shingles) vaccine	*Updated recommendations on the use of herpes zoster vaccines*	• Cost-utility analysis
2018	Pneumococcal vaccines	*Update on the use of pneumococcal vaccines in adults 65 years of age and older—A public health perspective*	• Cost-utility analysis
2019	Meningococcal vaccines	*The use of bivalent factor H binding protein meningococcal serogroup B (MenB-fHBP) vaccine for the prevention of meningococcal B disease*	• Review of economic studies
2022	RSV	*Recommended use of palivizumab to reduce complications of respiratory syncytial virus infection*	• Review of economic studies
2023	Pneumococcal vaccines	*Public health level recommendations on the use of pneumococcal vaccines in adults, including the use of 15-valent and 20-valent conjugate vaccines*	• Review of economic studies • Cost-utility analysis • Multi-model comparison
2024	Pneumococcal vaccines	*Recommendations for public health programs on the use of pneumococcal vaccines in children, including the use of 15-valent and 20-valent conjugate vaccines*	• Review of economic studies • Cost-utility analysis
2024	Pneumococcal vaccines	*Statement on the recommendations of the use of pneumococcal vaccines in adults, including PNEU-C-21*	• Review of economic studies • Cost-utility analysis
2024	HPV vaccines	*Updated recommendations on human papillomavirus vaccines*	• Review of economic studies
2024	RSV	*Statement on the prevention of respiratory syncytial virus disease in infants*	• Review of economic studies • Cost-utility analysis
2024	RSV	*Statement on the prevention of respiratory syncytial virus in older adults*	• Review of economic studies • Cost-utility analysis • Multi-model comparison
2024	Influenza vaccines	*Supplemental guidance on influenza vaccination in adults 65+*	• Review of economic studies
2024	COVID-19 vaccines	*Guidance on the use of COVID-19 vaccines during the fall of 2024*	• Cost-utility analysis

Abbreviations: HPV, human papillomavirus; RSV, respiratory syncytial virus

^a^ This table includes all NACI statements published between 2014 and 2024 that include formal economic evidence, such as systematic reviews and environmental scans, *de novo* cost-utility analyses, or multi-model comparisons. Statements that include general economic considerations were excluded

After exploring different formats for presenting economic evidence, NACI’s current approach involves posting a technical report to a preprint server to document the economic evidence considered during the committee’s deliberations, with key findings summarized in the NACI statement. These economic evaluations are often submitted for peer review at a later date and may evolve through that process.

### Membership

NACI has undergone important changes to membership to reflect the expanded needs of the committee. When EEFA and health economics were added to NACI’s mandate, PHAC created new voting member positions for two experts in pharmacoeconomics and one social scientist (medical anthropologist). Furthermore, during the COVID-19 pandemic, NACI selected a geriatrician to join the committee in order to provide perspectives on the increasing number of adult vaccines and formulations designed for older adults. This all resulted in a total membership of 16 (one Chair plus 15 other voting members, including the Vice-Chair).

Liaison organizations to NACI have also been updated over the last decade. NACI now includes liaison representatives from the Indigenous Physicians Association of Canada and the Canadian Indigenous Nurses Association to identify equity considerations and First Nations, Inuit and Métis healthcare perspectives, and from the Canadian Pharmacists Association, given the increasingly important role that pharmacists play in vaccine administration (**List 1**).

**List 1: Current liaison organizations to the National Advisory Committee on Immunization**

**Table:** 

Association of Medical Microbiology and Infectious Disease Canada Canadian Association for Immunization Research and Evaluation Canadian Immunization Committee Canadian Indigenous Nurses Association Canadian Nurses Association Canadian Paediatric Society Canadian Pharmacists Association Canadian Public Health Association Centers for Disease Control and Prevention (United States) The College of Family Physicians of Canada Council of Chief Medical Officers of Health Indigenous Physicians Association of Canada Society of Obstetricians and Gynaecologists of Canada

In the last ten years, there have been three NACI Chairs: Dr. Caroline Quach-Thanh (2017–2021), Dr. Shelley Deeks (2021–2023), and Dr. Robyn Harrison (2024–2025). All three played significant roles throughout the pandemic as Vice-Chair or Chair, overseeing the development of many COVID-19 updates to address the complex product environment in Canada. **Table 2** shows an updated list of all NACI Chairs since the committee’s formation in 1964. With the increased volume of NACI guidance due to public health emergencies and a rapidly expanding vaccine pipeline, together with concerted effort to meet the needs for timely complementary advice in relation to product authorizations, the workload of the NACI Chair has substantially increased.

**Table 2:** Chairs of the National Advisory Committee on Immunization

Years	Name
1964–1966	Dr. Andrew Rhodes, Toronto, ON (died February 1995)
1968–1969	Dr. Edward Bynoe (acting), Ottawa, ON (died March 2021)
1972–1989	Dr. J. Michael S. Dixon, Edmonton, AB (died November 2013)
1989–1993	Dr. Susan Tamblyn, Stratford, ON
1993–1998	Dr. David Scheifele, Vancouver, BC
1998–2003	Dr. Victor Marchessault, Ottawa, ON (died March 2003)
2003–2007	Dr. Monika Naus, Vancouver, BC
2008–2011	Dr. Joanne Langley, Halifax, NS
2011–2014	Dr. Bryna Warshawsky, London, ON
2014–2017	Dr. Ian Gemmill, Kingston, ON
2017–2021	Dr. Caroline Quach-Thanh, Montréal, QC
2021–2023	Dr. Shelley Deeks, Halifax, NS
2024–2025	Dr. Robyn Harrison, Edmonton, AB
2026–Present	Dr. Vinita Dubey, Toronto, ON

Abbreviations: AB, Alberta; BC, British Columbia; NS, Nova Scotia; ON, Ontario; QC, Québec

### Data submissions

Starting in 2020 during the COVID-19 pandemic, PHAC and NACI were afforded confidential direct access to the COVID-19 vaccine regulatory submission data, in accordance with subsection 21.1 (3) of the Food and Drugs Act and the Privacy Act ((12)). During the COVID-19 pandemic, access to regulatory materials allowed Health Canada and NACI to conduct parallel reviews, with several same-day decisions ((6)). Moving forward, however, the preferred model is sequential review. This approach ensures that NACI can draw on the full regulatory decision and product indications before issuing public health advice, and it also supports stakeholder discussions once the vaccine product monograph is publicly available. This allows for an efficient committee review process and will reduce the likelihood of unintentional off-label advice.

For new vaccines that are “same-in-class” or minor indication changes to old vaccines, NACI and PHAC are working on new expedited review pathways to ensure timely vaccine advice can keep pace with a growing pipeline of new vaccines. One such mechanism is a Canadian Immunization Guide (CIG) approvals board to facilitate a sub-structure of NACI to review and approve minor changes to advice in the CIG, accompanied by a summary of updates and evidence but without a full NACI statement. This will leverage clinical submission data from Health Canada, and NACI is currently piloting a clinical dossier submission process whereby vaccine manufacturers can submit a focussed data package directly to the NACI Secretariat to help launch the product reviews, which is not unlike submissions to Canada’s Drug Agency (CDA; formerly CADTH).

### Evidence reviews

The methods used by NACI have evolved over the last decade alongside international best practices for guidelines and the integration of the expanded program mandate elements in Canada. This includes integration of health economic evidence and EEFA factors (outlined above) and also implementation of the GRADE framework (Grading of Recommendations, Assessment, Development, and Evaluation), which is currently used by peer NITAGs, such as Germany, Australia, and the WHO’s Strategic Advisory Group of Experts on Immunization as well as by Canadian advisory committees, such as the Committee to Advise on Tropical Medicine and Travel (CATMAT) and the Canadian Task Force on Preventive Health Care (CTFPHC). With the increasing need for NACI to provide emergency guidance (e.g., COVID-19 and mpox) and a greater volume of statement updates due to a rapidly evolving product landscape, GRADE has not been possible or appropriate to use for every NACI statement.

While the GRADE methodology for immunization programs in its current format is not without its challenges, there is confidence that future collective and collaborative refinements by NITAGs can facilitate the desired aims to allow comparability, transparency, standardization and efficiency of complex public health decisions. By integrating GRADE methodology, where appropriate, in line with peer countries, it becomes possible to share and leverage each other’s work. This has already enabled sharing of otherwise time-consuming and labour-intensive systematic reviews so as to maximize efficiency. This includes recent NACI updates on Herpes Zoster program expansion to immunocompromised individuals 18 years and older and for optimal seasonal influenza product use in adults 65 years of age and older. NACI also looks towards use of artificial intelligence (AI) to accelerate evidence reviews as the body of scientific evidence continues to grow and in step with international consensus of best practices. There were early experiments by PHAC and NACI with AI during the pandemic ((13)) that are now being expanded in order to facilitate more rapid evidence collection and narrative summaries to provide subject matter experts with a timely evidence base to assess.

NACI continues to include fundamental vaccinology principles to guide decisions (e.g., extended intervals during COVID-19), in conjunction with published evidence as part of the GRADE process. NACI is often being asked to integrate evaluations for several products together at one time, such as RSV monoclonal antibody and maternal immunization program options. These complex program assessments can require integration of several parallel policy questions. To support this, NACI statements are being informed by PHAC evidence reviews of vaccine characteristics, burden of disease, or infectious disease modelling that are published or pre-printed separately in order to streamline the final NACI statements.

As described above in the overview of NACI’s expanded mandate, there was significant energy invested into developing a peer-reviewed framework for the systematic integration of EEFA factors into NACI guidance ((7)). The EEFA tools and supporting evidence are now applied routinely on NACI statements, and analyses of EEFA factors can be found in distinct sections of the NACI statements.

### Engagement with end-users, and collaborative guidance development

The world of guideline methods is also evolving to acknowledge the critical role for engagement of key populations and end-users in the guideline development process to inform assumptions about the acceptability of interventions, including vaccines. Historically, NACI would rely on input from liaison members representing different clinical practice groups to provide perspectives on behalf of their respective clinical groups and patient populations. NACI is now piloting models to engage directly with affected populations; for example, there was important direct engagement with a British Columbia organization representing sex workers, and the Ontario Gay Men’s Sexual Health Alliance (GMSHA) representing gay, bisexual, and men who have sex with men to understand perceptions of disease risk, vaccine acceptability, and strategies to prevent stigma during the mpox outbreak vaccine response in 2022 and 2023. Similarly, in 2024, NACI sought input on rabies vaccines from outdoor enthusiasts and occupational sectors who are most likely to be in contact with rabies-infected bats and animals. The committee has also considered questions from the Department of National Defence relevant to pre-deployment immunization for rabies protection (2018).

NACI is working with Indigenous health partners to establish a process that would better integrate First Nations, Inuit and Métis evidence and perspectives into the development of NACI statements; this will support national goals of reconciliation with Indigenous Peoples who have historically not been included in many health policy decisions, yet experience a high risk of vaccine-preventable illness due to social, environmental, and economic factors, rooted in the history of colonization and systemic racism in Canada. In 2021, the Indigenous Physicians Association of Canada and the Canadian Indigenous Nurses Association were added to NACI as formal liaison organizations. Informed by input from First Nations, Inuit and Métis health partners, NACI issued RSV statements in 2024 and several COVID-19 statements since 2021, including specific considerations for use in Indigenous communities where burden of illness may be higher based on intersecting structural and social determinants of health. NACI has reiterated in several guidelines that in First Nations, Inuit, and Métis communities, autonomous decisions should be made by Indigenous Peoples with the support of health care and public health partners in accordance with the *United Nations Declaration on the Rights of Indigenous Peoples Act*. Starting in 2026, NACI is launching a two-year pilot project to integrate an Indigenous health and immunization Working Group that will consider overarching principles of immunization for First Nations, Métis, and Inuit Peoples in Canada provide *ad hoc* engagement on specific recommendations for relevant vaccine preventable diseases (VPDs).

NACI and PHAC have found that building relationships with end-users and special population groups during periods between health emergencies is the preferred approach. This builds trust and communication channels that can be exercised during emergencies when guidance development happens much more quickly, leaving very narrow windows to identify and connect with affected populations groups during outbreaks.

### Global context

The international vaccine community continues to grow stronger through the Essential Programme on Immunization ((14)) and WHO’s Global NITAG Network. NACI has been working to collaborate through several mechanisms with other countries through both formal and informal approaches. During the COVID-19 pandemic, it became necessary to rely on and expand existing networks of NITAGs, and the research done in their supporting public health agencies. NACI received presentations from other countries such as the United Kingdom, United States, Israel, and Spain to inform decisions for COVID-19 vaccines. These informal networks continue to thrive based on regular touchpoints between several NITAG secretariats, creating a foundation for future rapid responses.

The work of NACI and PHAC has a growing international impact. In recent years, the CIG and NACI statements have been used by immunizers from all over the world. Although the majority of visits to the NACI and CIG web pages come from Canada (85%), both web pages have the same top five countries seeking our publications: United States, France, India, Ireland and the United Kingdom. Of note, traffic coming from outside of Canada to the French language content is significantly higher than to the English content, especially for the CIG webpages.

In 2024, PHAC and NACI launched a formal “NITAG twinning” initiative with the recently established NITAG for Haiti (GTCV-Haiti). This relationship, facilitated by the Pan American Health Organization (PAHO), resulted in productive exchanges and strategic support to decisions for the Haiti immunization program, despite the differences in committee maturity and country contexts.

Given the similar vaccine product environments and extensive connections and travel patterns between Canada and the United States, PHAC and NACI have a longstanding relationship with the Centers for Disease Control and Prevention (CDC) and the Advisory Committee on Immunization Practices (ACIP) in the United States. For the last several decades, there have been reciprocal liaison representatives across the two committees (NACI and ACIP). Each VPD working group has also historically included reciprocal technical leads from the respective committee secretariats who are able to share and provide insight into their respective country’s working group considerations and epidemiological contexts.

Global collaboration in addressing shared public health challenges has contributed to meaningful progress. For example, WHO guidance on HPV and rabies prompted immunization advisory committees worldwide, including NACI, to re-evaluate vaccine schedules and product access.

### NACI productivity

#### Outputs

**Figure 2:**
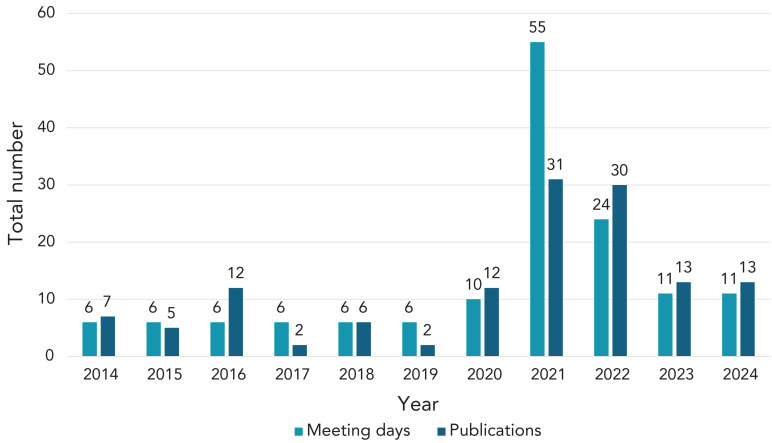
Number of National Advisory Committee on Immunization meetings and publications, 2014–2024

Throughout the COVID-19 pandemic, there was unprecedented interest in NACI’s immunization advice from PHAC, provinces and territories, and the general public. NACI was meeting weekly for much of the pandemic and issuing statements approximately every two weeks throughout the first years of vaccine rollout ((6)) (**Figure 2**).

Figure 2 illustrates how the number of NACI statements per year has risen from approximately four per year, surging to 10–30 per year during the pandemic and recent years. There have also been 12 to 20 CIG chapter updates per year in addition to four new chapters in the last decade. It is too soon to tell what the new normal will be in terms of outputs and frequency, but so far, the trend seems to be moving towards approximately 8 to 12 NACI statements per year. This will depend on the vaccine pipeline and also the secretariat resourcing available to support the committee over the next decade. **Table 3** shows the total number of publications by VPD from 2014 to 2024, with some VPDs having received multiple updates while others have not.

**Table 3:** National Advisory Committee on Immunization publications per vaccine preventable diseases, 2014–2024

Vaccine preventable diseases	Number of publications
COVID-19	67
Influenza	27
Pneumococcal	8
HPV	6
MMRV	5
Meningococcal	4
Diphtheria toxoid, tetanus toxoid & pertussis	3
Smallpox/mpox	3
Hepatitis	2
Herpes zoster	2
RSV	2
Ebola	1
Rotavirus	1

Abbreviations: HPV, human papillomavirus; MMRV, measles, mumps, rubella, and varicella; RSV, respiratory syncytial virus

While the primary target audience for NACI statements is made up of vaccine policy and program decision-makers, the primary audience for the CIG includes front-line immunizers (i.e., physicians, nurses and pharmacists) who look to the CIG for a pragmatic synthesis of NACI’s longer evidence-based guidelines. Visits to the CIG more than doubled in 2022 (1,487,642) compared to that of 2020 (702,405). It should be noted that the first version of the COVID-19 vaccine chapter was published on December 23, 2021; prior to this, all COVID-19 vaccine recommendations were only available in NACI statements. Traffic to the CIG last year (2024) was the highest on record at over two million visits. Interest in the CIG has grown steadily in the past decade with a spike in visits prompted by the COVID-19 pandemic. Looking at website visits over the past three years (since July 2022), the top five English language chapters were: COVID-19 vaccines; measles vaccines; recommended immunization schedules; pneumococcal vaccines; and RSV vaccines. Over the same time period, the top five French language chapters were: contraindications and precautions; herpes zoster (shingles) vaccine; measles vaccines; pneumococcal vaccines; and pertussis vaccines.

Prior to the pandemic, NACI web pages would attract approximately 250,000 visits annually. In 2021, with the publication of 27 COVID-19 related statements, traffic to NACI webpages expanded rapidly to over two million visits. Once COVID-19 vaccine guidance became available in a new CIG chapter (end of 2021), visits to the other NACI web pages dropped to just above pre-pandemic levels. Looking at the past three years (since July 2022), the top five English language NACI statements (in terms of visits) were: immunization in pregnancy with tetanus toxoid, reduced diphtheria toxoid and reduced acellular pertussis (Tdap) vaccine; statement on seasonal influenza vaccine for 2024–2025; guidance on the use of COVID-19 vaccines during the fall of 2024; public health level recommendations on the use of pneumococcal vaccines in adults, including the use of 15-valent and 20-valent conjugate vaccines; and guidance on an additional dose of COVID-19 vaccines in the spring for individuals at high risk of severe illness due to COVID-19. During the same period, the top five French language NACI statements were: Statement on seasonal influenza vaccine for 2023-2024; Statement on seasonal influenza vaccine for 2024–2025; Updated recommendations on the use of herpes zoster vaccines; Recommended use of palivizumab to reduce complications of respiratory syncytial virus infection in infants; and Public health level recommendations on the use of pneumococcal vaccines in adults, including the use of 15-valent and 20-valent conjugate vaccines.

## Discussion

### Knowledge translation

To improve knowledge translation, an email notification service is available to readers where they can subscribe to receive English or French email alerts with each new NACI publication or CIG chapter update (https://www.canada.ca/en/public-health/services/canadian-immunization-guide/subscribe.html). The publications mailing list now has over 11,300 English language subscribers and 750 French language subscribers. Interest in the subscription service has been increasing annually.

Another strategy to improve knowledge translation was to publish short rapid response interim statements starting in 2021 during the COVID-19 pandemic. For longer statements, PHAC published brief 1–2 page PHAC summaries of NACI’s advice online to provide a concise synopsis of the full technical document. The PHAC summaries now serve as a key tool to facilitate rapid public sharing of recommendations from NACI, sometimes receiving more web-visits than the source material in the full detailed NACI statements or rapid response statements.

### Growing vaccine pipeline

In the past five years, the number of new vaccine authorizations were more similar to what would be expected over a decade based on historical data from Health Canada. While some of this surge can be attributed to COVID-19 authorizations and strain updates, the vaccine pipeline also produced a flurry of activity around other pathogens, like *Streptococcus pneumoniae* and RSV, among others. Looking ahead, the pipeline of vaccine candidates in or entering phase 3 trials suggests a rapid and progressive expansion of the current vaccine landscape; notably we see a trend towards combination respiratory vaccines (e.g., COVID-19/influenza vaccines); vaccines targeted antimicrobial-resistant organisms (e.g., *Clostridioides difficile*, *Escherichia coli*, *Neisseria gonorrhoeae*); vaccines for special populations (e.g., cytomegalovirus, Group B *Streptococcus*); and new vaccines against vector-borne diseases (e.g., Lyme disease). In addition, improved vaccine formulations against some diseases, including COVID-19, influenza, and invasive pneumococcal disease are anticipated, as well as growth in novel vaccine technologies, particularly mRNA-based platforms.

As the development pipeline grows for new immunizing agents and therapeutic vaccines, PHAC has been working with the CDA to establish a triaging process to help determine whether post-authorization program reviews would be led by NACI or CDA when there is ambiguity. In recent years, PHAC has established a precedent of collaborating with CDA on health technology assessments for monoclonal antibodies to prevent COVID-19. This included an early assessment of the RSV monoclonal antibody nirsevimab ((15)), followed by a full assessment of the public health program for nirsevimab was conducted by NACI ((16)). Ongoing collaboration is anticipated to ensure that provinces and territories receive timely advice on each product, supporting informed decisions about funding through either public health budgets or drug plan budgets.

### Prioritization of vaccine programs for Canadians

There are now over 20 vaccine preventable diseases, multiple Health Canada authorized products for many of these diseases. There is also increasing attention on the use of immunization to prevent cancer (e.g., human papillomavirus related cancers; cancer related to hepatitis B), chronic diseases (e.g., cardiovascular disease prevention with influenza immunization; prevention of dementia with herpes zoster vaccine), and to improve health system efficiencies by reducing the need for access to acute care, medical transport, outbreaks, and costs through primary infection prevention.

However, the rapidly increasing number of vaccine products is juxtaposed against relatively static public health budgets which makes prioritization more important than ever. In Canada, as in other high-income countries, spending on publicly funded immunization programs is estimated to account for well under 1% of overall health care expenditures ((17,18)). Prioritization is now identified internationally as a necessity, and various tools are being proposed ((19)).

Over the last decade, the Canadian process for NACI work plan prioritization has been conducted on a one- or two-year cycle led by the NACI Secretariat at PHAC. There is structured engagement with the provinces and territories (through the CIC and the Council of Chief Medical Officers of Health—CCMOH), engagement with NACI committee members and liaison organisations to conduct ranking of potential workplan topics ahead of and during the two years of the workplan.

A challenge for the decade ahead will be prioritizing the work to gain the most meaningful impacts in the short and long term. Refocusing some priorities on the protection of specific population groups (such as adults, pregnant women and pregnant people, those with frailty, equity deserving individuals, or those at key life stages) may be one path forward, rather than evaluating individual vaccine products in isolation. The committee will also need to continue balancing the tension between predictable and timely advice for each newly authorized product, versus more complex, multi-product comparisons addressing the optimal strategy for a population.

### Reconciliation with First Nations, Inuit, and Métis Peoples

The most recent Interim National Immunization Strategy (2025–2030) identified that NACI and PHAC should continue to work on new and improved engagement models for key populations and that: “*NACI considers populations at higher risk of disease or severe outcomes when formulating guidance. When appropriate to the VPD and epidemiology, NACI guidance is developed engaging with immunization experts from First Nations, Inuit and Métis health systems, as well as from populations at higher risk of VPDs or severe outcomes of VPDs where appropriate. NACI guidance development integrates Canadian specific considerations related to ethics, equity, feasibility, and acceptability, as well as cost-effectiveness.*“

The committee has benefitted greatly from the ability to consult a Vaccine Preventable Disease Working Group convened by Indigenous Services Canada (ISC) in recent years to complement the Indigenous Physicians Association of Canada, the Canadian Indigenous Nurses Association, and ISC representation at NACI meetings and the review of NACI statements. There is still much work to be done to build trusting relationships to improve pathways to vaccine access for First Nations, Inuit and Métis Peoples, and to further ensure representation and integration of First Nations, Inuit and Métis perspectives into NACI guidance. The shared goal is to improve health through meaningful reconciliation, achieved through collaborative efforts.

In the methods section above, additional ongoing collaborations between NACI and Indigenous partners have been highlighted.

## Conclusion

### Challenges new and old

Affordability, sustainability, and equity are positioned at the forefront as challenges now and going forward. While it is increasingly clear that vaccine programs are often cost-effective and can be cost-saving, challenges of affordability and budget impact remain. Although cost is not NACI’s primary focus, rising vaccine prices and limited budgets inevitably influence the committee’s work, as well as the collective prioritization of the immunization and funding communities. Fully capturing the long-term economic and societal benefits of vaccination will require new collaborations and improved data to strengthen the case for vaccines as essential public health investments and to ensure their value is fully reflected in funding and policy decisions.

In conclusion, the opportunities and challenges of the past decade have enabled the already mature NACI to continue to build upon the same initial and founding strengths of NACI in 1964. Over the years, renewed speed and efficiency, enhanced methodologies, and the benefit of additional representation and engagement from key experts and partners in Canada has incrementally expanded the scope and capacity of NACI. This could not be done without the strong foundation and trust that had been built thanks to the service of each of the dedicated NACI participants and leaders over the six preceding decades. The last decade was facilitated by a skilled secretariat, the addition of the expertise required to onboard cost-effectiveness analyses, a focus on equity promotion, improvements in global and domestic vaccine research and development, and vaccine-preventable disease surveillance in Canada. These factors and this foundation, together with the dedicated and expert volunteer committee members, keep the committee moving forward into what might be the most dynamic decade yet ahead.
